# Soil aggregates stability and storage of soil organic carbon respond to cropping systems on Black Soils of Northeast China

**DOI:** 10.1038/s41598-019-57193-1

**Published:** 2020-01-14

**Authors:** Meng Zhou, Chunzhu Liu, Jie Wang, Qingfeng Meng, Ye Yuan, Xianfa Ma, Xiaobing Liu, Yingxue Zhu, Guangwei Ding, Jizhou Zhang, Xiannan Zeng, Weiling Du

**Affiliations:** 1 School of Resources and Environment, Northeast Agricultural University, Harbin, 150030 China; 2 Northeast Institute of Geography and Agroecology, Chinese Academy of Sciences, Harbin, 150081 China; 3 Department of Biological Center, Harbin Academy of Agricultural Sciences, Harbin, 150028 China; 4 Mudanjiang Tobacco Science Rsearch Institute, Heilongjiang Branch of China Tobacco Corporation, Harbin, 150076 China; 5 Chemistry Department, Northern State University, Aberdeen, SD 57401 USA; 6 Institute of Natural Resources and Ecology, Heilongjiang Academy of Sciences, Harbin, 150040 China; 7 Institute of Crop Cultivation and Tillage, Heilongjiang Academy of Agricultural Sciences, Harbin, 150086 China

**Keywords:** Environmental impact, Chemical physics

## Abstract

Monoculture and improper management may reduce soil fertility and deteriorate soil structure in Black soils (Mollisols) of Northeast China. The experiment was carried out from 2015 to 2016 in Black Soils comprising five cropping systems: continuous corn (CC), soybean-corn rotation (SC), corn-soybean rotation (CS), fallow-corn (FC), and fallow-soybean (FS). Our results showed that CS and FS treatments significantly increased mean weight diameter (MWD) and fractal dimension (D) in mechanical stability aggregates (MSAs), and increased MWD and geometric mean diameter (GMD) in water-stable aggregates (WSAs) compared with CC treatment. These two treatments were also significantly increased water-stable aggregates stability rate (WSAR), but decreased percentage of aggregates destruction (PAD) than CC treatment. Meanwhile, CS and FS treatments exhibited a higher carbon accumulation than CC treatment in bulk soils. Soil organic carbon (SOC) concentration in WSA_0.106-0.25_,WSA_2-5 mm_ and WSA_0.5-1 mm_ had a dominant effect on aggregate stability. Simutaneously, SOC in WSA_>5 mm_ affected SOC concentration in bulk soils. As a whole, the CS and FS treatments can increase the percentage of macro-aggregates, enhance aggregate stability, as well as increase SOC concentration in bulk soils and all soil aggregate sizes.

## Introduction

Soil organic carbon (SOC) plays a key role in forming and stabilizing soil structure, enhancing soil physical properties, and nutrient recycling^1–3^. Soil aggregate, the basic unit of soil structure, mediates many physical and chemical processes in soils^4–8^, such as soil compaction, soil nutrient recycling, soil erosion, root penetration, and crop yield^9^. Aggregate stability is frequently used as an indicator of soil structure^10–12^ because better soil structure and higher aggregate stability are vital to improve soil fertility, soil sustainability, and productivity^13,14^.

SOC influenced aggregate stability and soil structure^15,16^. The stability of organic carbon in different size aggregates is different. Organic carbon in the micro-aggregates is less susceptible to change than it is in the macro-aggregates^17^. The soil organic matters of different cropping systems differed based on the quantity and quality of the crop residue coverage and the environment, affecting the organic carbon contents of the soil and the aggregate stability^18^. The cropping systems mainly create conditions for the decomposition and transformation of soil organic matter by changing the distribution of soil organic carbon and the active habitat of microorganisms, thereby causing changes in soil aggregates^19^.

Soil mean weight diameter (MWD), geometric mean diameter (GMD), fractal dimension (D), percentage of aggregates destruction (PAD) and water-stable aggregates stability rate (WSAR) are all indicators of soil aggregate stability. The larger the MWD and GMD values are, the higher the average particle size agglomeration of soil aggregates are, and the stronger the stability of soil structure is^20^. Castrignano and Stelluti^21^ found that the larger the D value of soil aggregates are, the easier dispersed the aggregates were. The amount of soil micro-aggregates (WSA_0.053-0.25 mm_) increases with the increase of D value^22^. The lower the PAD value and higher WSAR value are, the more stable the soil structure is, and the better the soil erosion resistance is, and thus the higher the soil fertility is^23,24^.

Legume crops with non-legume crop rotation extensively applied around the world are important technologies for conserving soil resources and achieving sustainable development of agriculture^25–30^. The two-year rotation system of corn-soybean increased soil fertility, adjusted the balance of nutrients in the soil, and controlled soil degradation in the cold region^31^. Compared with continuous cropping, leguminous-graminaceous crop rotations were able to increase the farmland biodiversity with time growing^32^. Rotation in conjunction with manure amendment management decelerated the degradation of water stable macro-aggregates with re-aggregated structure^33^.

To conserve soil resources, land fallow practices have been implemented in many parts of the world. The Conservation Reserve Program (CRP) was proposed by the United States in 1986^34^, and this measures can control soil erosion^35^, improve water quality^36^, protect biodiversity^37^, create the habitat for wild animals and plants, and ameliorate agricultural non-point source pollution^38^. Thus, the experience is worth referencing with respect to land reclamation and land protection^39–41^.

Black Soils in Northeast China, also referred to as Mollisols or Chernozems, are one of the most precious soil resources for sustainable agricultural development in the country^33^, which play an irreplaceable role in safeguarding China’s regional eco-environmental security and national food security^42,43^. The original Black Soils demonstrate a high fertility and excellent physicochemical characteristics with approximately 50–80 g kg^−1^ SOC^44,45^. However, the wealth of agricultural Black Soils has undergone a decline in natural fertility and soil structure deterioration due to long-term continuous cropping and monoculture as well as excessive reclamation^46^.

The main cropping systems in Northeast China are continuous cropping and crop rotation with different crop sequences. Previous studies indicated that long-term tillage affected the formation and stabilization of soil aggregate^10,12,14,47–49^, which holds SOC stocks reckoned as an important strategy in maintaining soil structure and enhancing the condition of soil particles^9,50^. However, limited information is available on the impact of rotation and intermittent fallow on the distribution and aggregate stability in Black Soils of Northeast China. We rationally hypothesized that cropping systems with rotation and intermittent fallow will possibly affect the processes of aggregate formation. The objectives of this study were (1) to determine the distribution and stability of mechanical stability aggregates (MSAs) water-stable aggregates (WSAs) under different cropping systems in Black Soils; (2) to evaluate cropping impact on the SOC concentrations and stocks in bulk soils and WSAs; and (3) to understand how the cropping systems influence the correlations between the parameters of soil structure stability and WSAs, as well as SOC concentrations.

## Material and Methods

### Experimental site

The study was conducted from 2015 to 2016 at the research farm of Northeast Agricultural University, located at the Xiangyang, Harbin, China (longitude 126°54′8.68″E, latitude 45°46′14.27″N, and altitude 184 meters). Before 2015, our experimental site was planted corn all the time. The site is in a moderate temperate zone with a semi-humid continental monsoon climate. The mean annual temperature is 3.5 °C with a frost-free period of 142 days, and the mean annual precipitation is 553.9 mm. The soil type in the area is typical Black Soils with light loam texture. Soil physic-chemical properties were as following: soil pH of 6.8; soil organic matter (SOM) of 35 g kg^−1^, total nitrogen (TN) of 1.71 g kg^−1^, available nitrogen (AN), phosphorus (AP), and potassium (AK) of 76 mg kg^−1^, 12 mg kg^−1^, and 158 mg kg^−1^, respectively, and bulk density (ρ_b_) of 1.24 g cm^−3^.

### Experimental design and management

The experimental treatments for two consecutive years were as follows:

Continuous corn (CC): Corn was planted with hole sowing in the spring of 2015 and intertillaged with 15–20 cm soil depth in the mid-June of 2015, and then chopped corn stalk was incorporated into the soil with 25–30 cm plowing depth at harvest in the autumn of 2015. The tillage methods of 2016 was the same as 2015.

Soybean-corn rotation (SC): Soybean was planted with drill sowing in the spring of 2015 and intertillaged with 10–15 cm soil depth in the mid-June of 2015, and then chopped soybean stalk was incorporated into the soil with 25 cm plowing depth at harvest in the autumn of 2015. Next, corn was planted on the original ridge with hole sowing in the spring of 2016 and intertillaged with 15–20 cm cm soil depth in the mid-June of 2016, and then chopped corn stalk was incorporated into the soil with 25–30 cm plowing depth at harvest in the autumn of 2016.

Corn-soybean rotation (CS): Corn was planted with hole sowing in the spring of 2015 and intertillaged with 25 cm soil depth in the mid-June of 2015, and then chopped corn stalk was incorporated into the soil with 30–35 plowing depth at harvested in the autumn of 2015. Next, soybean was planted on the original ridge with drill sowing in the spring of 2016 and intertillaged with 15–20 cm soil depth in the mid-June of 2016, and then chopped soybean stalk was incorporated into the soil with 25–30 cm plowing depth at harvest in the autumn of 2016.

Fallow-corn (FC): The experimental site was abandoned without any tillage measures and restored by natural vegetation in 2015. Next, corn was planted with hole tillage in the spring of 2016 and intertillaged with 15–20 cm soil depth in the mid-June of 2016, and then chopped corn stalk was incorporated into the soil with 25–30 cm plowing depth at harvest in the autumn of 2016.

Fallow-soybean (FS): The experimental site was abandoned without any tillage measures and restored by natural vegetation in 2015. Next, soybean was planted with drill tillage in the spring of 2016 and intertillaged with 15–20 cm soil depth in the mid-June of 2016, and then chopped soybean stalk was incorporated into the soil with 25–30 cm plowing depth at harvest in the autumn of 2016.

The experimental design was a randomized complete block with three replications consisting of 15 plots in total. Each plot was 10.0 meters long and 3.9 meters wide with row spacing of 0.65 m. The soybean (*Glycine max*) variety was Jiannong No. 18 (Soybean Institute of Jilin Academy of Agricultural Sciences of China) and the corn (*Zea mays*) variety was Xianyu 335 (Pioneer Corporation Ltd., United States). For fertilization, the compound fertilizer (N-P_2_O_5_-K_2_O:15-23-10) was applied at a rate of 225 kg ha^−1^ year^−1^ as the base fertilizer of soybean; and the compound fertilizer (N-P_2_O_5_-K_2_O: 23-12-13) was applied at a rate of 375 kg ha^−1^ year^−1^ as the base fertilizer and urea was applied at a rate of 150 kg ha^−1^ year^−1^ on jointing stage of corn according to the amount of fertilizer application after soil testing in the local area.

### Soil sampling

All soil samples (0–20 cm) were collected from each plot in the harvest of 2016. Undisturbed soil samples with dimensions of 20 cm (height) × 30 cm (length) × 30 cm (width) were obtained using a spade from five points by “S” type in each plot for the determination of WSAs. Composite bulk soil samples were collected from the same plots for the measurement of SOC and TN concentration in bulk soil.

### Laboratory method

The distribution and stability of soil aggregates were measured according to the dry sieving and wet sieving method created by Elliott^51^. The specific dry sieving method was as follows. 200-gram samples of soil were passed through a series of six sieves (5, 2, 1, 0.5, 0.25, and 0.106 mm) to isolate seven aggregate size fractions and shaked them gently. Meanwhile, stones, roots and other impurities were eliminated in the soil samples of each size. Then the whole series of sieves were moved up and down for 7 minutes at 30 cycle min^−1^ manually. Finally, the mass of dry sieve aggregates with different particle sizes was measured and weighed as *W*_*di*_.

At the same time, the specific wet sieving method was as follows. 50-gram samples of soil were air-dried for 24 hours and evenly distributed over the nested sieve surfaces through a series of six sieves (5, 2, 1, 0.5, 0.25, and 0.106 mm) to isolate seven aggregate size fractions. The nest was set at the highest point when the oscillation cylinders were filled with distilled water. Soil samples were completely covered with water. To slake the air-dried soil, 1 L of distilled water was rapidly added to each cylinder until the soil sample and top screen were covered with water. The soils were submerged in water for 10 minutes before the start of the wet-sieving action. The apparatus specifications of oscillation time (10 minutes), stroke length (4 cm vertical) and frequency (30 cycle min^−1^) were held constant. Material remaining on each sieve was collected, dried at 60–80 degrees Celsius, and weighed as *M*_1_. The water-stable aggregate distribution was based on the percentage of total mass in each aggregate fraction.

The soil particles remaining on the sieve were dried and weighed as *M*_2_, (Eq. 1). The mass of each graded aggregate *M*_0_ was calculated from Eq. (1).

1 $${M}_{0}={M}_{1}-{M}_{2}$$

The aggregate fraction in each sieve represented the water-stable aggregates (WSAs) according to class size. WSA_>2 mm_, WSA_0.25-2 mm_, and WSA_0.053-0.25 mm_ were considered as the large macro-aggregates, small macro-aggregate, and micro-aggregates^4,52,53^, respectively.

*W*_*i*_ is the proportion of each aggregate class in relation to the weight of the soil samples (Eq. 2). Wet-sieving is calculated from Eq. (3).

2 $${W}_{i}=\frac{{W}_{di}}{200}\times 100 \% $$

3 $${W}_{i}=\frac{{M}_{o}}{50}\times 100 \% $$

According to the data of each aggregate class obtained by the experiment, R_0.25_ means aggregates larger than 0.25 mm in diameter, the mean weight diameter (MWD)^54^, and geometric mean diameter (GMD)^55^ are computed as Eqs. (4), (5), and (6), respectively.

4 $${R}_{0.25}=\frac{{M}_{r > 0.25}}{{M}_{T}}=1-\frac{{M}_{r < 0.25}}{{M}_{T}}$$

5 $$MWD=\mathop{\sum }\limits_{i=1}^{n}(\overline{{X}_{i}}{W}_{i})$$

6 $$GMD=\exp (\frac{\mathop{\sum }\limits_{i=1}^{n}Wi\,{\rm{lg}}\,\overline{Xi}}{\mathop{\sum }\limits_{i=1}^{n}Wi})$$

*X*_*i*_ is the mean diameter of the class (mm). M(r < *X*_*i*_) means the mass of aggregates, which are smaller than *Xi*. *M*_*T*_ means total mass of the aggregates.

The fractal dimension^56^ is computed as Eq. 7:

7 $$(3 \mbox{-} D){\rm{lg}}(\frac{\overline{di}}{{d}_{{\rm{\max }}}})=\,{\rm{lg}}[\frac{{W}_{(\delta \le \overline{di})}}{{W}_{0}}]$$

Take ${\rm{lg}}[\frac{{W}_{(\delta \le \overline{di})}}{{W}_{0}}]$ and ${\rm{lg}}(\frac{\overline{di}}{{d}_{{\rm{\max }}}})$ as the horizontal and vertical axis, respectively. It draws the conclusion that 3-D is the slope of straight line between ${\rm{lg}}(\frac{\overline{di}}{{d}_{{\rm{\max }}}})$ and ${\rm{lg}}[\frac{{W}_{(\delta \le \overline{di})}}{W0}]$. Soil fractal dimension is marked as D.

The formula for calculating the percentage of aggregates destruction and water-stable aggregates stability rate were calculated as seen Eqs. (8) and (9).

8 $$PAD=\frac{{M}_{d}-{M}_{w}}{{M}_{d}}\times 100 \% $$

9 $$WSAR=\frac{WSA}{A}\times 100 \% $$

where PAD is the percentage of aggregates destruction (%), *M*_*d*_ and *M*_*w*_ represent the aggregate mass fractions of dry sieve and wet sieve with >0.25 mm particle sizes, respectively. WSAR is the water-stable aggregates stability rate (%), WSA and A mean the water-stable aggregate weight (g) and mechanical stability aggregate weight (g), respectively.

Composite bulk soil samples for each treatments with three replications were also air dried at room temperature (22 degrees Celsius), passed through a 0.25 mm diameter sieve, and stored at room temperature prior to the analysis of SOC concentrations. SOC in the bulk soils and WSAs were determined by 0.8 mol L^−1^ 1/6 K_2_Cr_2_O_7_ oxidation and FeSO_4_ titration^57^. Soil available phosphorus and potassium (AP and AK) were measured by 0.5 mol L^−1^ NaHCO_3_ and molybdenum antimony anti-colorimetric method, and 1 mol L^−1^ CH_3_COONH_4_ extraction method, respectively^57^. The SOC stock calculated using the following Eq. (10)^58^.

10 $${{\rm{M}}^{\prime} }_{{\rm{i}}}={{\rm{C}}}_{{\rm{i}}}\times {{\rm{SOC}}}_{{\rm{i}}}\times {\rm{BD}}\times {\rm{H}}\times {10}^{-1}$$

where, M′_i_ means SOC stock of i-level aggregates (t hm^−2^), C_i_ and SOC_i_ mean relative mass fraction and SOC concentration of i-level aggregates, respectively. BD means soil bulk density in the 0–20 cm (g cm^−3^), H is the thickness of soil layer and we take it as 20 cm in our research.

### Statistical analysis

All statistical analysis was carried out using SPSS (Statistical Package for Social Science) 20.0 for Windows and all graphs were drawn using Origin 7.5. Significant differences among treatments for MSAs, WSAs, SOC concentrations and stocks, as well as MWD, GMD, D, PAD, and WSAD were determined with one-way analysis of variance in the different treatments with the same aggregate sizes followed by the least significant difference (LSD) test at *P* < 0.05 (n = 9). The correlations among the measured soil attributes were determined using stepwise regression analysis^59^.

## Results

### Size distribution of mechanical stability aggregates

MSAs were mainly concentrated in MSA_>5 mm_, ranging from 35.4% in SC treatment to 50.2% in FC treatment (Table 1). The lowest aggregate content was found in the MSA_<0.106 mm_, accounting for about 2%. The highest proportions in MSA_>5 mm_, MSA_2-5 mm_, and MSA_1-2 mm_ were obtained in FS (50.2%), FC (24.8%), and FC (14.6%) treatments, respectively. Meanwhile, we were surprised to find that SC treatment documented the highest proportion in the MSA_0.5-1 mm_ (17.4%), MSA_0.25-0.5 mm_ (6.5%), MSA_0.106-0.25 mm_ (2.9%), and MSA_<0.106 mm_ (2.3%). On the other hand, the lowest proportions in the MSA > 5 mm and MSA_2-5 mm_ were identified in FC treatment (34.7%) and CC treatment (18.5%), respectively. While, the FS treatment had the lowest proportions in the MSA_1-2 mm_ (11%), MSA_0.5-1 mm_ (11.8%), MSA_0.25-0.5 mm_ (1.9%), MSA_0.106-0.25 mm_ (0.8%), and MSA_<0.106 mm_ (1.2%).

**Table 1:** Distribution of soil mechanical stability aggregate size fractions (percentage) under different cropping systems.

Treatments	Aggregate size (percentage)						
	MSA_>5 mm_	MSA_2-5 mm_	MSA_1-2 mm_	MSA_0.5-1 mm_	MSA_0.25-0.5 mm_	MSA_0.106-0.25 mm_	MSA_<0.106 mm_
CC	45.02 ± 0.52 b	18.46 ± 0.32 d	11.70 ± 0.11 bc	15.01 ± 0.21 b	5.84 ± 0.05 b	2.07 ± 0.04 b	1.90 ± 0.04 a
SC	35.42 ± 0.48 c	21.91 ± 0.27 bc	13.58 ± 0.15 ab	17.44 ± 0.19 a	6.49 ± 0.07 a	2.92 ± 0.03 a	2.26 ± 0.07 a
CS	43.58 ± 0.67 b	20.31 ± 0.22 c	12.38 ± 0.13 bc	14.20 ± 0.08 b	5.31 ± 0.08 c	2.02 ± 0.02 b	2.20 ± 0.06 a
FC	34.68 ± 0.41 c	24.82 ± 0.16 a	14.63 ± 0.18 a	15.98 ± 0.18 ab	5.58 ± 0.06 bc	2.26 ± 0.03 b	2.06 ± 0.05 a
FS	50.22 ± 1.03 a	23.16 ± 0.18 ab	11.02 ± 0.09 c	11.81 ± 0.15 c	1.86 ± 0.03 d	0.76 ± 0.01 c	1.16 ± 0.02 b

**Note:** CC, SC, CS, FC, and FS represent continuous corn, soybean-corn rotation, corn-soybean rotation, fallow-corn, and fallow-soybean treatments. MSA indicates mechanical stability aggregate. MSA_>5 mm,_ MSA_2-5 mm,_ MSA_1-2 mm,_ MSA_0.5-1 mm,_ MSA_0.25-0.5 mm,_ MSA_0.106-0.25 mm_ and MSA_<0.106 mm_ represent > 5 mm, 2–5 mm, 1–2 mm, 0.5–1 mm, 0.25–0.5 mm, 0.106–0.25 mm and <0.106 mm aggregate fractions in mechanical stability aggregate. Values are given as mean ± standard error (n = 9). Mean value ± SE in the same column followed by the different lowercase letters indicate significant differences among different treatments for each size of soil aggregates (L.S.D. test, *P* < 0.05).

Compared with CC treatment, SC treatment significantly (*P* < 0.05) increased the proportion in MSA_2-5 mm_, MSA_0.5-1 mm_, MSA_0.25-0.5 mm_, and MSA_0.106-0.25 mm_ by 18.7%, 16.2%, 11.1%, and 41.1%, respectively. CS treatment only significantly (*P* < 0.05) increased the proportion in MSA_2-5 mm_ by 10%. However, FC treatment both significantly (*P* < 0.05) increased the proportion in MSA_2-5 mm_ and MSA_1-2 mm_ by 34.5% and 25%. Simultaneously, FS treatment significantly (*P* < 0.05) increased the proportion in MSA_2-5 mm_ and MSA_>5 mm_ by 11.6% and 25.5%, respectively.

### Size distribution of water-stable aggregates

After the two-year experiment, we found that among the five treatments, the highest proportions in the WSA_>5 mm_, WSA_2-5 mm_, WSA_1-2 mm_ and WSA_0.5-1 mm_ were obtained in SC (4.3%), FS (7.6%), FC (9.3%), and CS (20.7%) treatments, respectively (Table 2). However, the lowest proportion in the WSA_>5 mm_ was the FC treatment (0.3%), and The lowest proportions in WSA_2-5 mm_, WSA_1-2 mm_ and WSA_0.5-1 mm_ were all found in CC treatment with 3.9%, 2.9%, and 8.9%, respectively. We were surprised to note that the CC treatment documented the highest proportion in the WSA_0.25-0.5 mm_ (20.6%), WSA_0.106-0.25 mm_ (30.5%), and WSA_<0.106 mm_ (31.2%). On the other hand, the lowest proportions in the WSA_0.25-0.5 mm_, WSA_0.106-0.25 mm_, and WSA_<0.106 mm_ were identified in CS (13.9%), FS (18.7%), and CS (22.1%) treatments, respectively. The FS treatment showed the highest proportion in the WSA_>0.25 mm_ (55.2%), followed by CS (51.9%), SC (49.3%), and FC (47.7%) treatments (Table 2). The CC treatment only had 38.4% in WSA_>5 mm_.

**Table 2:** Distribution of soil water-stable aggregate size fractions (percentage) under different cropping systems.

Treatments	Aggregate size (percentage)						
	WSA_>5 mm_	WSA_2-5 mm_	WSA_1-2 mm_	WSA_0.5-1 mm_	WSA_0.25-0.5 mm_	WSA_0.106-0.25 mm_	WSA_<0.106 mm_
CC	2.04 ± 0.05 c	3.90 ± 0.04 d	2.90 ± 0.02 e	8.94 ± 0.37 d	20.59 ± 1.15 a	30.46 ± 1.04 a	31.17 ± 1.13 a
SC	4.31 ± 0.06 a	6.27 ± 0.07 b	3.85 ± 0.03 d	14.88 ± 0.62 c	19.99 ± 0.83 a	22.05 ± 0.94 c	28.66 ± 1.10 ab
CS	2.07 ± 0.03 c	6.45 ± 0.06 b	8.83 ± 0.06 b	20.70 ± 0.48 a	13.85 ± 0.45 b	26.02 ± 0.66 b	22.09 ± 0.57 c
FC	0.27 ± 0.01 d	5.80 ± 0.04 c	9.27 ± 0.07 a	13.82 ± 0.62 c	18.55 ± 0.64 a	23.60 ± 0.54 c	28.69 ± 1.09 ab
FS	3.39 ± 0.05 b	7.57 ± 0.05 a	7.01 ± 0.08 c	16.77 ± 0.54 b	20.49 ± 0.59 a	18.72 ± 0.88 d	26.05 ± 0.61 b

**Note:** CC, SC, CS, FC, and FS represent continuous corn, soybean-corn rotation, corn-soybean rotation, fallow-corn, and fallow-soybean treatments. WSA indicates mechanical stability aggregate. WSA_>5 mm,_ WSA_2-5 mm,_ WSA_1-2 mm,_ WSA_0.5-1 mm,_ WSA_0.25-0.5 mm,_ WSA_0.106-0.25 mm_ and WSA_<0.106 mm_ represent > 5 mm, 2–5 mm, 1–2 mm, 0.5–1 mm, 0.25–0.5 mm, 0.106–0.25 mm and < 0.106 mm aggregate fractions in water-stable aggregate. Values are given as mean ± standard error (n = 9). Mean value ± SE in the same column followed by the different lowercase letters indicate significant differences among different treatments for each size of soil aggregates (L.S.D. test, *P* < 0.05).

Compared with the CC treatment, the SC and FS treatments significantly (*P* < 0.05) increased the proportion in WSA_>5 mm_ by 111.3% and 66.2%, respectively. The SC, CS, FC, and FS treatments all significantly (*P* < 0.05) increased the proportion of WSA_2-5 mm_ (60.8%, 65.4%, 48.7% and 94.1%), WSA_1-2 mm_ (32.8%, 204.5%, 219.7% and 141.7%), and WSA_0.5-1 mm_ (66.4%, 131.5%, 54.6% and 87.6%), respectively. However, all the treatments significantly (*P* < 0.05) decreased the proportion of WSA_0.106-0.25 mm_. The proportion of WSA_<0.106 mm_ was significantly (*P* < 0.05) decreased in the CS and FS treatments by 29.1% and 16.4%, respectively.

### MWD, GMD, D, PAD, and WSAR within soil aggregates

From the point of MSAs, the highest MWD and lowest D were both obtained in the CS treatment with 4.84 and 2.03, respectively (Table 3). Meantime, the lowest MWD and highest D were both found in the CC treatment, respectively (Table 3). In comparison to CC treatments, SC, CS, and FS treatments all significantly (*P* < 0.05) increased MWD with 13.5%, 27.7%, and 14.5%; and decreased D with 1.4%, 7.7%, and 1.4%, respectively (Table 3). However, no significant (*P* < 0.05) difference was found for GMD values among the five treatments.

**Table 3:** MWD, GMD, D, PAD and WSAR under different cropping systems in MSAs and WSAs.

Treatments	MWD		GMD		D		PAD (%)	WSAR (%)
	MSAs	WSAs	MSAs	WSAs	MSAs	WSAs		
CC	3.79 ± 0.03 c	0.55 ± 0.02 c	1.00 ± 0.02 a	0.21 ± 0.01 c	2.20 ± 0.01 a	2.86 ± 0.02 a	60.05 ± 0.82 a	40.03 ± 0.54 e
SC	4.30 ± 0.02 b	0.84 ± 0.03 a	1.00 ± 0.01 a	0.28 ± 0.01 b	2.17 ± 0.02 b	2.83 ± 0.01 a	48.02 ± 0.97 c	51.56 ± 0.46 c
CS	4.84 ± 0.02 a	0.78 ± 0.02 b	1.01 ± 0.02 a	0.33 ± 0.02 a	2.03 ± 0.01 c	2.79 ± 0.02 a	45.82 ± 0.75 d	54.47 ± 0.62 b
FC	3.83 ± 0.04 c	0.59 ± 0.01 c	1.00 ± 0.01 a	0.27 ± 0.03 b	2.19 ± 0.03 a	2.83 ± 0.03 a	50.14 ± 0.66 b	49.24 ± 0.58 d
FS	4.34 ± 0.05 b	0.87 ± 0.04 a	1.00 ± 0.01 a	0.32 ± 0.02 a	2.17 ± 0.02 b	2.80 ± 0.02 a	43.69 ± 0.81 e	57.92 ± 0.41 a

**Note:** CC, SC, CS, FC, and FS represent continuous corn, soybean-corn rotation, corn-soybean rotation, fallow-corn, and fallow-soybean treatments. Soil properties of MWD, GMD, D, PAD, and WSAR indicate mean weight diameter, geometric mean diameter, fractal dimension, percentage of aggregates destruction, and water-stable aggregates stability rate, respectively. MSAs and WSAs indicate mechanical stability aggregates and water-stable aggregates. Values are given as mean ± standard error (n = 9). Mean value ± SE in the same column followed by the different lowercase letters indicate significant differences among different treatments (L.S.D. test, *P* < 0.05).

From the point of WSAs, the highest MWD and GMD were observed in the FS and CS treatments, respectively, while the lowest MWD and GMD were both characterized in the CC treatment (Table 3). Compared with the CC treatment, the SC, CS, and FS treatments significantly (*P* < 0.05) increased MWD and GMD by 52.7%, 41.8%, and 58.2%; and by 33.3%, 57.1%, and 52.4%, respectively. The maximum and minimum D were obtained in the CC and CS treatments (Table 3). However, no significant (*P* < 0.05) difference was observed for D values among the five treatments.

The PAD declined and WSAR increased in the order of CC < FC < SC < CS < FS treatment (Table 3). In comparison with CC treatment, the FC, SC, CS, and FS treatments significantly (*P* < 0.05) decreased PAD and increased WSAR by 16.5%, 20%, 23.7%, and 27.2%, and by 23%, 28.8%, 36.1%, and 44.7%, respectively.

### SOC concentrations and stocks within water-stable aggregate sizes and bulk soils

**Figure 1:**
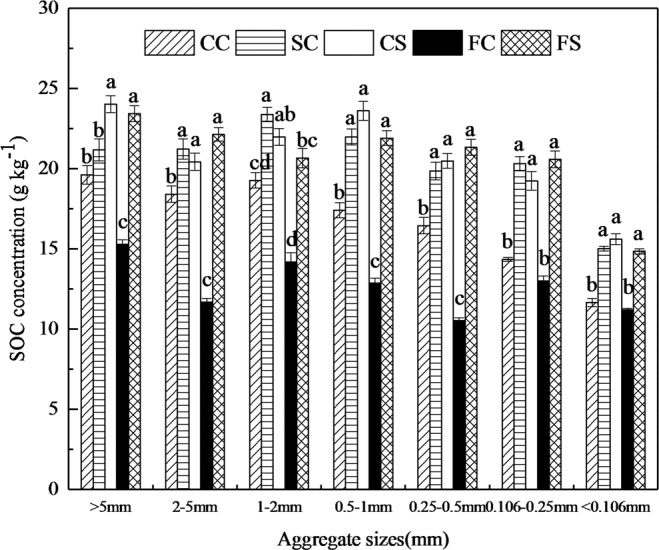
Soil organic carbon (SOC) concentration in the soil aggregate sizes under different treatments. Values are given as mean ± standard error (n = 9). Different lowercase letters indicate significant differences among different treatments for each size of soil aggregates (L.S.D. test, *P* < 0.05). CC, SC, CS, FC, and FS represent continuous corn, soybean-corn rotation, corn-soybean rotation, fallow-corn, and fallow-soybean treatments, respectively.

**Figure 2:**
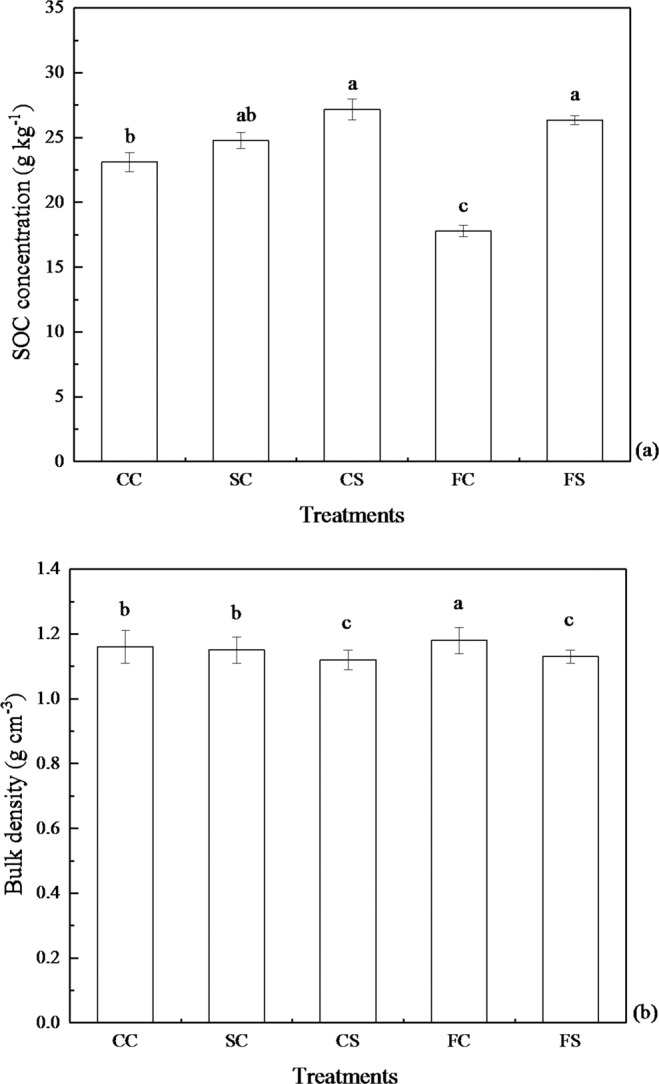
SOC concentration (**a**) and bulk density (**b**) in bulk soils under different treatments. SOC indicates soil organic carbon. CC, SC, CS, FC, and FS represent continuous corn, soybean-corn rotation, corn-soybean rotation, fallow-corn, and fallow-soybean treatments, respectively (n = 9).

In general, compared with the CC treatment, SC treatment increased SOC concentration in each water-stable aggregate size and bulk soils, while, the FC treatment decreased SOC concentration in the WSAs (Fig. 1) and bulk soils (Fig. 2a). Specifically, the SC treatment had the highest SOC concentration in the WSA_1-2 mm_ with 23.38 g kg^−1^ (Fig. 1). The highest SOC concentrations in the WSA_>5 mm_, WSA_0.5-1 mm_, and WSA_<0.106 mm_ were obtained by the CS treatment with 24.02 g kg^−1^, 23.61 g kg^−1^, and 15.60 g kg^−1^, respectively (Fig. 1). Meanwhile, the highest SOC concentrations in the WSA_2-5 mm_, WSA_0.25-0.5 mm_, and WSA_0.106-0.25 mm_, WSAs were obtained in the FS treatment with 22.13 g kg^−1^, 21.33 g kg^−1^, and 20.58 g kg^−1^, respectively (Fig. 1).

The SOC concentration in the WSA_2-5 mm_, WSA_0.5-1 mm_, WSA_0.25-0.5 mm_, WSA_0.106-0.25 mm_, and WSA_<0.106 mm_ were all significantly (*P* < 0.05) increased by 15.2%, 26.2%, 20.7%, 41.6%, and 28.7% from SC treatment; by 11%, 35.6%, 24.5%, 34.2%, and 33.8% from CS treatment; and by 20.2%, 25.8%, 29.7%, 43.5%, and 27.4% from FS treatment in comparison with the CC treatment, respectively (Fig. 1). Simultaneously, compared with CC treatment, CS and FS treatments both significantly (*P* < 0.05) increased SOC concentration in the WSA_>5 mm_ by 22.4% and 19.4%, as well as SC and CS treatments both significantly (*P* < 0.05) increased SOC concentration in the WSA_1-2 mm_ by 21.4% and 14.1%, respectively (Fig. 1). In addition, the CS and FS treatments both significantly (*P* < 0.05) increased SOC concentration by 17.6% and 14.1% compared with the CC treatment in bulk soils (Fig. 2a).

**Figure 3:**
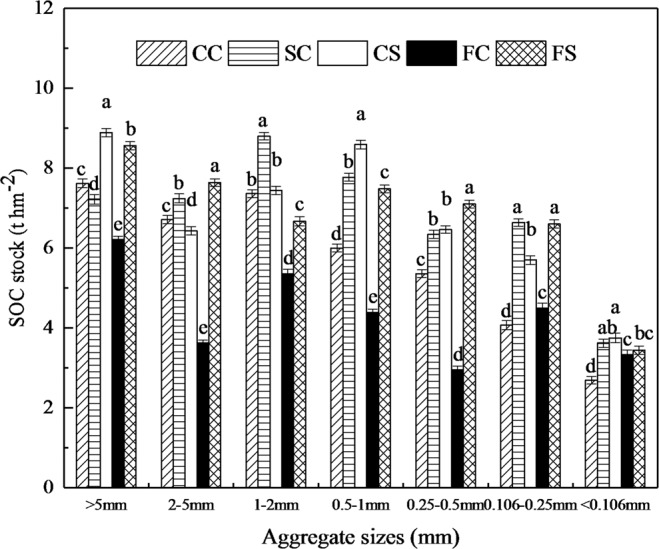
Soil organic carbon (SOC) stocks in the soil aggregate sizes under different treatments. Values are given as mean ± standard error (n = 9). Different lowercase letters indicate significant differences among different treatments for each size of soil aggregates (L.S.D. test, *P* < 0.05). CC, SC, CS, FC, and FS represent continuous corn, soybean-corn rotation, corn-soybean rotation, fallow-corn, and fallow-soybean treatments, respectively.

Across all treatments, the SOC stock in the seven aggregates’ sizes showed a similar tendency in the SOC concentration, although the bulk density differed a little among the treatments (Fig. 2b). Bulk density for the five treatments was in the range of 1.12–1.18 g cm^−3^ (Fig. 2b). CS treatment had the highest SOC stock in the WSA_>5 mm_, WSA_0.5-1 mm_, and WSA_<0.106 mm_ with 8.89 t hm^−2^, 8.59 t hm^−2^, and 3.75 t hm^−2^, respectively (Fig. 3). While the FS treatment had the highest SOC stock in the WSA_2-5 mm_ (7.64 t hm^−2^), WSA_0.25-0.5 mm_ (7.10 t hm^−2^), respectively (Fig. 3). Furthermore, the SC treatment demonstrated the biggest SOC stock in the WSA_1-2 mm_ (8.80 t hm^−2^) and WSA_0.106-0.25 mm_ (6.64 t hm^−2^), respectively (Fig. 3). Except for WSA_0.106-0.25 mm_ and WSA_<0.106 mm_, the FC treatment documented the lowest SOC stock in all five other aggregate sizes.

Furthermore, the SOC stock in the WSA_2-5 mm_, WSA_1-2 mm_, WSA_0.5-1 mm_, WSA_0.25-0.5 mm_, WSA_0.106-0.25 mm_, and WSA_<0.106 mm_ from the SC treatment, the stock in the WSA_>5 mm_, WSA_0.5-1 mm_, WSA_0.25-0.5 mm_,WSA_0.106-0.25 mm_, and WSA_<0.106 mm_ from the CS treatment were all significantly (*P* < 0.05) increased by 8%, 19.6%, 29.5%, 18.3%, 63.1%, and 34.7%; and by 16.7%, 43.1%, 20.6%, 40.2%, and 39.4% compared with CC treatment, respectively (Fig. 3). Similarly, the SOC stock in the WSA_0.106-0.25 mm_ and WSA_<0.106 mm_ from the FC treatment, and the stock in the WSA_>5 mm_, WSA_2-5 mm_, WSA_0.5-1 mm_,WSA_0.25-0.5 mm_, WSA_0.106-0.25 mm_, and WSA_<0.106 mm_ from the FS treatment were also significantly (*P* < 0.05) increased by 10.7%, and 23.8%; and by 12.3%, 13.8%, 24.7%, 32.4%, 62.3%, and 27.9 in comparison with the CC treatment, respectively (Fig. 3).

**Figure 4:**
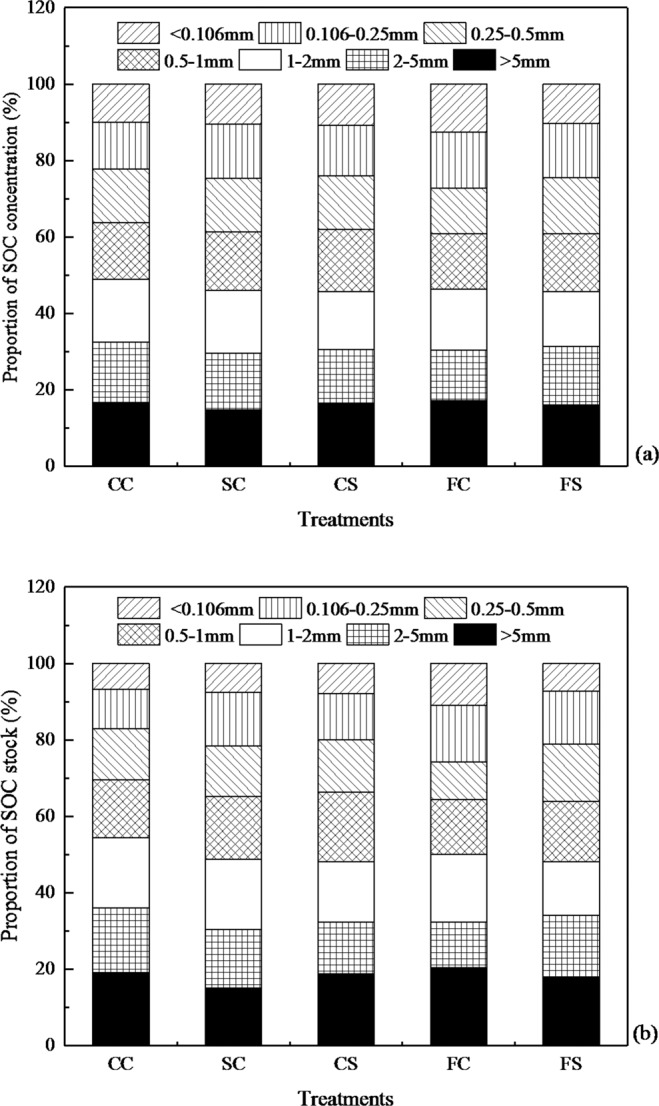
Proportion of SOC (**a**) and SOC stock (**b**) in the soil aggregate sizes under different treatments. SOC indicates soil organic carbon. CC, SC, CS, FC, and FS represent continuous corn, soybean-corn rotation, corn-soybean rotation, fallow-corn, and fallow-soybean treatments, respectively (n = 9).

On the whole, the SOC concentration and stocks in WSAs both accounted for almost half of the total in small macro-aggregates (WSA_0.25-2 mm_) under the five cropping systems (Fig. 4a,b). For instance, SOC concentrations and stocks were 45.6% and 48.1% in the SC treatments; 45.5% and 47.6% in the CS treatments; and 44.1% and 44.7% in the FS treatment (Fig. 4a,b).

### Correlations among measured soil attributes and parameters

Linear regression models between measured soil attributes and parameters, obtained by stepwise regression analysis, were listed in Table 4. The data indicated that the MWD in WSAs was significantly and positively correlated to SOC concentration in WSA_0.106-0.25 mm_, as shown in Eq. (10) (*R*^2^ = 0.942; *P* = 0.006). Similarly, significant and positive correlation was obtained between SOC concentrations in bulk soils and SOC in the WSA_>5 mm_, as shown in Eq. (11) (*R*^2^ = 0.988; *P* = 0.001). Simultaneously, significant correlations were obtained between the GMD and D in WSAs and WSAs of each particle size. In specification, significant and positive correlations were exhibited between GMD in WSAs and WSA_2-5 mm_, and WSA_0.5-1 mm_, as shown in Eq. (12) (*R*^2^ = 0.936; *P* = 0.007). However, a significant and negative correlation was demonstrated between D in WSAs and WSA_0.5-1 mm_, as shown in Eq. (13) (*R*^2^ = 0.940; *P* = 0.006).

**Table 4:** Relationship among soil aggregate stability in WSAs and SOC in WSAs, soil aggregate stability in WSAs and WSAs, as well as SOC concentration in bulk soils and WSAs of all water-stable aggregate size fractions under five treatments.

Indicators in Y	Indicators in X	Regression model (Equation number)	R^2^	F	P
Aggregate stability (MWD) in water-stable aggregates	SOC in water-stable aggregate particle sizes	Y_MWD_ = 0.069 SOC_WSA 0.106-0.25 mm_ + 0.025 (10)	0.942	49.123	0.006
SOC in bulk soils	SOC in water-stable aggregate particle sizes	Y_SOC_ = 1.814 SOC_WSA> 5 mm_ + 2.036 (11)	0.988	255.224	0.001
Aggregate stability (GMD) in water-stable aggregates	water-stable aggregate particle sizes	Y_GMD_ = 0.014 WSA_2-5 mm_ + 0.007 WSA_0.5-1 mm_ + 0.088 (12)	0.936	43.656	0.007
Aggregate stability (D) in water-stable aggregates	water-stable aggregate particle sizes	Y_D_ = −0.006 WSA_0.5-1 mm_ + 2.916 (13)	0.940	46.777	0.006

Note: Eq. (10) *Y*_*MWD*_ MWD value in WSAs; SOC_*WSA 0.106-0.25* mm_ SOC in water-stable aggregate 0.106–0.25 mm size fraction.

Eq. (11) *Y*_*SOC*_ SOC in bulk soils; *SOC*_*WSA> 5* mm_ SOC in water-stable aggregate >5 mm size fraction.

Eq. (12) *Y*_*GMD*_ GMD value in WSAs; *WSA*_*2-5* mm_ water-stable aggregate 2–5 mm size fraction; *WSA*_*0.5-1* mm_ water-stable aggregate 0.5–1 mm size fraction.

Eq. (13) *Y*_*D*_ D value in WSAs; *WSA*_*0.5-1* mm_ water-stable aggregate 0.5–1 mm size fraction.

MWD, GMD, D, SOC and WSAs represent mean weight diameter, geometric mean diameter, fractal dimension, soil organic carbon and water-stable aggregates, n = 45.

## Discussion

In the perspective of size distribution, the proportion of macro-aggregates with MSAs under different cropping systems in our experiment were significantly higher than that of WSAs, which may be due to the fact that the soil is more and more compacted with the increasing of agricultural mechanization operations^60–62^.

Water stable aggregates were the indicator of soil anti-disintegration^63^, and the aggregate sizes elucidated the effects of management on soil structural stability. Relevant studies demonstrated that soil aggregates were divided into macro-aggregates (WSA_>0.25 mm_) and micro-aggregates (WSA_<0.25 mm_)^53,64^. Six *et al*.^65^ believed that macro-aggregates were the best structures in the soil, and the higher the content, the better agglomeration and stablity of soil aggregates.

In the current study, the proportion of WSA_>0.25 mm_ was in the order of FS > CS > SC > FC > CC treatments, which suggested that fallow and rotation were more conducive to the formation of WSA_>0.25 mm_ than continuous corn. Higher microbial biomass in the rhizosphere with soybean or rotation might be responsible for the formation of macro-aggregates^66^. Alternatively, Nakamoto and Suzuki^67^ held a different opinion. They indicated that the root growth of soybean illustrated a stronger destructive effect on macro-aggregates in the soil. Planting soybean could reduce the number of macro-aggregates compared with corn even though the root density of soybean was relatively small. Small macro-aggregates (WSA_0.25-2 mm_) were found to be the leading size fractions in the SOC concentrations and stocks in our study, which was consistent with other findings^68,69^.

In the present study, fallow-corn and soybean-corn rotations were significantly greater than continuous corn in MWD and GMD, while D was smaller in the CS and FS treatments than the CC treatment. Our findings indicated that fallow-corn and soybean-corn rotations had made the soil aggregate structure more stable. Soil aggregates stability is expressed by MWD of the size range, which is proportional to the amount of larger WSAs^70^. Devine *et al*.^71^ in Horseshoe Bend of USA showed that no-tillage increased MSAs. Furthermore, MWD in WSAs compared with conventional tillage, MWD increased with the increasing of soil depths.

Many studies believed that MWD was related to soil organic matter content. Das *et al*.^70^ found that the increased amounts of macro-aggregates (WSA_>2 mm_) for applying green manure, cereal residues, and farmyard manure on wheat and rice were associated with MWD. The correlation of strong linearity between MWD and SOC suggested that SOC content in the WSA_0.106-0.25 mm_ played a major role in soil aggregates stability.

In this study, the four treatments of soybean-corn, corn-soybean, fallow-corn, fallow-soybean all had lower PAD and higher WSAR than that of continuous corn treatment, which indicated that the effect of rotation and intermittent fallow practices on soil aggregates stability was more favorable than planting crops. Six *et al*.^64^ had shown that tillage accelerated the renewal rate of macro-aggregates, which was not conducive to the formation of micro-aggregates in macro-aggregates. The reduction of tillage disturbance increased the stability of soil aggregates^19,72,73^, which may be because the frequent tillage of farmland destroys the soil particle structure, increases the soil aeration, and deteriorates the protection of soil particles, resulting in loose soil structure and increase damage to the soil structure^74^.

Our experiment found that the stability of soil aggregates treated by corn-soybean and soybean-corn in both legumes and gramineae rotation system was higher than continuous corn treatment, which may be because that the rotation of legumes and gramineae plants could rapidly increase the storage of soil organic carbon and promote the formation of macro-aggregates and stabilization of micro-aggregates^75^. We conclude that the rotation system can increase the energy required for microbial life activities in the soil, produce cementing substances that form soil aggregates, weaken the destruction of soil aggregates, and reduce the damage to soil structure.

In our research, MWD and D in MSAs demonstrated that CS treatment had the best soil aggregate stability. However, MWD in WSAs as well as PAD and WSAR showed that FS treatment had the highest soil aggregate stability. GMD in WSAs showed the highest stability was corn-soybean treatment, but our study found that corn-soybean and fallow-soybean treatments were not significantly different. These five indicators indicate that the effects of soil aggregate stability are basically similar, and they can be mutually verified with each other. Therefore, we concluded that CS and FS treatment had higher soil aggregate stability combining these five indicators.

The SOC contents in soil aggregates of each particle size is a microscopic characterization between soil organic matter balance and mineralization rate, which has dual significance in soil fertility and soil carbon sink^76^. Our research found that SOC concentration exhibited the “M” type among the seven particle sizes in that the WSA_1-2 mm_ and WSA_0.106-0.25 mm_, had higher SOC concentration in the five different cropping systems. Furthermore, through the regression models, we obtained that WSA_2-5 mm_ and WSA_0.5-1 mm_ dominated the primary particle sizes in soil structure stability. Therefore, we speculated that the small macro-aggregates (WSA_0.25-2 mm_) had larger SOC concentration accumulation and higher soil aggregates stability. These results corresponded with Puge’s viewpoint that macro-aggregates were a source of organic carbon enrichment^77,78^. Tisdall and Oades^79^ proposed that the formation and stability of micro-aggregates and macro-aggregates were interrelated processes. A wealth of research had manifested that macro-aggregates composed of organic binding agents^80–84^. Consequently, the protective mechanism of macro-aggregates on SOC was better than micro-aggregates^85,86^.

However, some researchers had the distinctive standpoints, for instance, Christensen^87^, De Jonge *et al*.^88^, and Li *et al*.’s^89^ research demonstrated that organic carbon was mainly distributed in micro-aggregates (WSA_<0.25 mm_) and that organic carbon contents increased with the decrease of aggregate particle size’s decrease. Li *et al*.^69^ found that organic carbon is distributed in a “V” shape in aggregates as the result of the organic carbon contents of aggregates d > 2 mm and d < 0. 25 mm are high.

In our study, CS and FS treatments did show more C accumulation compared with CC treatment in bulk soils, which might be beneficial to the formation of soil structure and the enhancement of soil structure stability^90^. Alternatively, the formation of aggregates affects the decomposition of SOC^4^. Huang *et al*.^91^ believed that the factors affecting soil aggregates all influenced soil carbon, while the quantity and quality of soil carbon were closely related to aggregates. Meng *et al*.^92^ showed that the main reason for the decline of aggregate stability and the decrease of water-stable aggregates were the reduction of soil organic matter.

It was interesting to illustrate in this study that the SOC concentration in fallow-soybean treatment was higher than continuous corn, and fallow-corn treatments in the WSA_>5 mm_, WSA_2-5 mm_, WSA_0.25-0.5 mm_, and WSA_0.106-0.25 mm_. This might be due to the accelerated mineralization of SOC in the soil with corn stalk, which acts as a cementing material to facilitate the formation of macro-aggregates^93^.

## Conclusions

Small macro-aggregates (WSA_0.25-2 mm_) dominated the concentration of SOC in the five cropping systems. SOC concentration in the WSA_0.106-0.25 mm_, WSA_2-5 mm_, and WSA_0.5-1 mm_ had a dominant effect on aggregate stability as well as SOC in WSA_>5 mm_ affected SOC concentration in bulk soils. Corn-soybean and fallow-soybean can increase macro-aggregates, enhance aggregate stability, and increase SOC concentrations in bulk soils and all aggregate sizes. These results are likely related to decrease the soil aeration with no tillage in farmland, and rapidly increasing the storage of SOC with the rotation of legumes and gramineae plants, and then reinforcing soil structure and promoting stabilization of soil aggregates.
